# Prevalence of Substance Use Among Students in Health Colleges in Saudi Arabia: A Cross-Sectional Study

**DOI:** 10.7759/cureus.70890

**Published:** 2024-10-05

**Authors:** Khaldoon Aljerian, Rahaf Alamri, Asma Althomali, Lama Aljamili, Lujain Alkhalaf, Zahra Alsultan, Shuliweeh Alenezi, Mohammed Alarabi

**Affiliations:** 1 Pathology, King Saud University, Riyadh, SAU; 2 Psychiatry, King Saud University, Riyadh, SAU

**Keywords:** college, cross-sectional studies, prevalence, saudi arabia, substance abuse, substance misuse

## Abstract

Background: Substance misuse is a global and substantial health concern that includes the inappropriate use of illicit substances or prescription and over-the-counter medications, which subsequently leads to poor outcomes such as a mental health disorder, financial distress, work malfunction, and academic failure.

Aim: This study aimed to assess the prevalence of substance use, identify associated risk factors, explore potential complications within this specific demographic, and investigate the relationship between sociodemographic/lifestyle variables such as age, gender, marital status, family income, smoking, physical activity, and academic variables including grade point averages (GPAs), study hours, and the parental educational level with substance use history among students in health colleges in Saudi Arabia.

Methods: This quantitative, descriptive cross-sectional study employed a questionnaire-based approach to collect data from students in health colleges across the regions of Saudi Arabia in the year 2022.

Results: The study found the prevalence of the use of various substances among students in health colleges to be 20.2%. Multiple factors were found to be associated with substance abuse. Smoking habits were the strongest predictor of substance misuse, with 49.1% of smokers reporting a history of substance use, compared to only 15.5% of non-smokers. This makes smoking the most impactful lifestyle factor related to substance use. Sleep patterns also played a significant role. A notable 43.6% of those who took up to two hours to fall asleep reported substance use, followed by 32.4% of those with sleep disorders and 25.5% with poor sleep quality. Unpredictable sleep patterns and a history of sleep disorders further strengthened the link between disrupted sleep and substance misuse. Academic performance, specifically GPA, showed that individuals with lower academic achievement were more likely to report substance use. Among those with a GPA below 3.0, 43.5% had a history of substance use, while only 16.7% of those with a GPA above 4.5 did so.

Conclusions: The findings underscored the high prevalence of substance use, even among students in health colleges, and shed light on its connection to specific determinants. This poses a serious public health concern, as substance use is associated with poorer GPA, diminished knowledge acquisition, and overall academic performance. This, in turn, may negatively impact their future roles as healthcare providers, compromising both the quality of patient care and their personal well-being. These results emphasize the need for further investigations to unravel the underlying mechanisms of substance misuse and formulate innovative strategies to address this pressing issue effectively.

## Introduction

Substance misuse is a serious public health problem worldwide. It is defined as the inappropriate use of illegal substances or over-the-counter or prescription medications, which can potentially lead to significant harm to the users or those around them. Substance misuse may be occasional or more frequent and may lead to the development of substance use disorders [1]. Worldwide studies show that substance misuse in late adolescence and young adulthood is a growing concern that is associated with significant health burdens, such as mental health issues, academic decline, physical health consequences, and social consequences.

The period between 18 and 25 years of age is a transitional and stressful period as it is marked by instability due to changes in education, living arrangements, and relationships. It is also marked by biological and developmental changes, in which the brain continues to develop and mature. This period of vulnerability coincides with the age of entry into college or university. Also, some evidence was found for emotional intelligence (EI) as a possible factor in mediating stress; however, the results did not determine the direction of this relationship [2].

A recent study investigated the link between EI and health risk behaviors, including substance use, among university students. The study found that higher levels of EI were associated with lower impulsivity and reduced sensitivity to immediate rewards, which in turn lowered the likelihood of engaging in risky behaviors such as substance misuse. This research highlights that EI plays a key role in managing impulsive tendencies, helping individuals make more thoughtful decisions in stressful situations, thereby reducing the risk of substance misuse [3].

In order to address this concern, researchers have recently explored the rates of substance use and misuse among college students in the region [4-6]. Nevertheless, there is a paucity of literature on this topic in the Gulf Cooperation Council countries. In Saudi Arabia, certain substances such as stimulants, khat, alcohol, opioids, water pipes, and cigarettes have been reported to be used [7], given that COVID-19 might have had an impact on the misuse of these substances among at-risk college students [8]. The objectives of this study are to measure the prevalence, associated risk factors, and complications of substance misuse among students in health colleges in Saudi Arabia.

Understanding how educational environments influence substance use is crucial. Previous studies have highlighted how variations in educational settings can affect prescription analgesic misuse [9]. Similarly, it is important to examine how different aspects of the educational environment, such as institutional culture and academic pressures, may impact substance use among students in health colleges in Saudi Arabia.

It is important to study the prevalence of substance use and its associated factors. Our aim in this cross-sectional study was to estimate the prevalence of substance use among students in health colleges and explore which lifestyle- or education-related factors strongly predicted substance use.

## Materials and methods

The study utilized an anonymous self-administered questionnaire to obtain descriptive cross-sectional data on substance use among students in health colleges in Saudi Arabia. The time period was from March 2022 until December 2022. Ethical approval for this study was obtained from the Institutional Review Board of King Saud University (approval number: E-22-7066, dated 29 August 2022). All participants provided informed consent prior to their inclusion in the study. We used a validated questionnaire for the assessment of substance use, which was incorporated into an online survey after obtaining the author’s consent. This study used a convenient sampling technique through the distribution of an online Google Docs survey (Google LLC, Mountain View, CA, USA) to collect data from the target population through social media platforms. The survey was completed by 391 healthcare college students from all regions of Saudi Arabia. The questionnaire was comprised of 34 questions covering the following sections: socio-demographics, lifestyle, sleep quality and disorders, education, academic performance, and lifetime history of substance use. Our inclusion criteria were being a healthcare college student and living in Saudi Arabia.

Statistical analysis

The statistical analysis was conducted using SPSS Statistics version 28.0 (IBM Corp. Released 2021. IBM SPSS Statistics for Windows, Version 28.0. Armonk, NY: IBM Corp.). Continuous variables are described using means and standard deviations (SD), with the comparison of two means tested via a Student's t-test. Categorical variables are presented as percentages, with the correlation between them assessed using the chi-square test for independence. To identify significant explanatory variables (predictors) for our outcome variable, which is reported substance use, we constructed a multivariable binary logistic regression. The significance of the relationship between explanatory variables (predictors) and the outcome variable is described using the p-value, odds ratio (OR), and the 95% confidence interval (CI) of the OR. The model's accuracy percentage, the model goodness of fit (Hosmer-Lemeshow goodness of fit), and the pseudo-R-square will be reported. In all tests, the level of significance is set at a p-value <0.05.

## Results

Our sample included 391 students whose ages ranged from 18 to 30 years. The majority of respondents (271, 69.3%) were female. Among the surveyed 391 students in health colleges, 312 (79.8%) reported no history of substance use. Table 1 shows the frequency of various substances used by the 79 students who reported substance use.

**Table 1:** Reported substance misuse among students in health colleges in Saudi Arabia Data are presented in frequency and percentage

Reported substance	N	%
Stimulants	20	5.1
Opiates and opioids	12	3.1
Khat	20	5.1
Hypnotics	13	3.3
Sedative drugs	41	10.5
Hallucinogens	8	2.0
Alcohol	5	1.3
Dissociative anesthetics	4	1.0
Cannabinoid	3	0.8
New synthetic agents	3	0.8
inhalants drugs	1	0.3
Alternative alcohol	3	0.8
Others	4	1.0
None of these	312	79.8

We analyzed the frequency of reported substance use based on sociodemographic variables and lifestyle factors. As shown in Table 2, smoking habits were the only lifestyle variable significantly associated with a history of substance use. Fifty-two (15.5%) of non-smokers reported a history of substance use compared to 27 (49.1%) of smokers.

**Table 2:** Sociodemographic and lifestyle variables and substance misuse Level of significance is p<0.05. Data are presented in frequency and percentage SAR: Saudi riyal

Variable		Substance misuse				p-value
		No		Yes		
		N	%	N	%	
Age in years	18-25	305	80.3	75	19.7	Insufficient
	>25	7	63.6	4	36.4	
Gender	Male	95	79.2	25	20.8	0.84
	Female	217	80.1	54	19.9	
Marital status	Single	305	80.5	74	19.5	Insufficient
	Married	5	55.6	4	44.4	
	Divorced	2	66.7	1	33.3	
Smoking	Non-smoker	284	84.5	52	15.5	<0.01
	Smoker	28	50.9	27	49.1	
Exercise regularly	Yes	102	75.6	33	24.4	0.13
	No	210	82	46	18	
Family income	Less than 20,000 SAR	162	77.1	48	22.9	0.14
	20,000 SAR and more	135	83.3	27	16.7	

We analyzed the relationship between reporting substance use and education-related variables, including grade point averages (GPAs), study hours, and the parent’s educational level. As shown in Table 3, students with lower GPAs had a higher proportion of respondents reporting substance use.

**Table 3:** Education-related variables and substance misuse Level of significance is p<0.05. Data are presented in frequency and percentage

Variable		Substance misuse				p-value
		No		Yes		
		N	%	N	%	
Academic year	Preparatory	56	73.7	20	26.3	0.41
	1st/2nd	103	79.2	27	20.8	
	3rd-5th year	120	82.2	26	17.8	
	Intern/postgrad	33	84.6	6	15.4	
GPA (out of 5)	<3	13	56.5	10	43.5	0.03
	3-3.99	48	76.2	15	23.8	
	4-4.49	53	79.1	14	20.9	
	4.5+	125	83.3	25	16.7	
Study hours per day	<2 hours	54	74.0	19	26.0	0.3
	2-3 hours	55	83.3	11	16.7	
	4-5 hours	93	85.3	16	14.7	
	6-8 hours	74	77.1	22	22.9	
	>8 hours	36	76.6	11	23.4	
Fathers’ education	High school/below	108	78.8	29	21.2	0.88
	University graduated	137	79.7	35	20.3	
	Post-graduate	67	81.7	15	18.3	
Mothers’ education	High school/below	123	79.9	31	20.1	0.19
	University graduated	156	82.1	34	17.9	
	Post-graduate	33	70.2	14	29.8	

Tables 4-5 show the self-reported justifications for substance use and the perceived harmful consequences, respectively. The most frequently reported justifications for substance use were peer pressure and mental health reasons. Remarkably, the majority of respondents did not believe that their substance use was associated with any harmful consequence.

**Table 4:** Self-reported justifications for substance misuse Data are presented in frequenci and percentage

Reported justifications	N	%
Mental health disorder	17	21.5
Peer pressure	33	41.8
Drug availability in the community	11	13.9
Joy seeking	10	12.7
Academic failure or lack of academic motivation	9	11.4
Anti-social behaviour, including early aggressive behaviour	5	6.3
Family history of addiction	11	13.9
Lack of family involvement	8	10.1
Poverty	4	5.1

**Table 5:** Reported harmful consequences after substance misuse Data are presented in frequency and percentage

Consequences	N	%
None	71	89.9
Financial problems	1	1.3
Suicidality	3	3.8
Poor performance at work	6	7.6
Anxiety or paranoid thinking	3	3.8
Complicated grief	2	2.5
Difficulty concentrating or remembering	1	1.3
Changes in behavior	1	1.3
Slowed reaction time	2	2.5
Neglected appearance	2	2.5
Physical health issues	2	2.5

We have studied the relationship between sleep-related variables and substance use in our sample of college students, as shown in Table 6. Sleep variables included reported sleep quality, sleep hours per day, the time needed to fall asleep, sleeping patterns, and a history of sleep disorders. Having an unpredictable sleep pattern, taking longer to fall asleep, and having a history of a sleep disorder were all associated with reporting substance use.

**Table 6:** Sleep variables and substance misuse Level of significance is p<0.05. Data are presented in frequency and percentage

Variable		Substance misuse				p-value
		No		Yes		
		N	%	N	%	
Sleep quality	Poor	38	74.5	13	25.5	0.5
	Average	78	81.3	18	18.8	
	Good	117	78.0	33	22.0	
	Excellent	79	84.0	15	16.0	
Sleep disorders	No	187	90.8	19	9.2	<0.01
	Yes	125	67.6	60	32.4	
Sleep hours per day	<6 hours	72	74.2	25	25.8	
	6-8 hours	168	81.2	39	18.8	
	>8 hours	72	82.8	15	17.2	
How much time do you need to fall asleep	Few minutes	112	81.2	26	18.8	<0.01
	Up to half/one hour	155	82.9	32	17.1	
	Up to two hours	22	56.4	17	43.6	
	More than two hours	23	85.2	4	14.8	
Sleeping pattern	Night and day	101	82.1	22	17.9	<0.01
	Night only	124	84.4	23	15.6	
	Day only	4	33.3	8	66.7	
	Unpredictable	83	76.1	26	23.9	

We used a multivariate binary logistic regression model in order to explore which variables were significantly associated with increased odds of substance use, as shown in Table 7. The outcome variable was a lifetime history of substance use, whereas the potential predictor variables included all significantly associated variables from the bivariate analysis. The results of our logistic regression analysis show that the strongest predictor was smoking, with an OR of 6.29 (95% CI: 2.69-14.68, p<0.01). Another significant predictor was having a history of sleep disorders; students reporting sleep disorders had 4.25 times higher odds of lifetime substance misuse than those who did not (OR: 4.25, 95% CI: 2.06-8.77, p<0.01). Additionally, the odds of reporting lifetime substance use decreased by 14% as the family size increased by one member (OR: 0.86, 95% CI: 0.74-0.99, p=0.04). The Hosmer-Lemeshow test for goodness of fit was significant at 0.71. The pseudo-R-square (Nagelkerke R square) was 0.29, suggesting that the model explains 29% of the variance in reported lifetime substance use.

**Table 7:** Multivariate binary logistic regression results of substance misuse Hosmer-Lemeshow test for goodness of fit was significant at 0.71, pseudo-R-square at 0.29 (Nagelkerke R square), and overall model accuracy at 83.2% (specificity = 97.5%%, sensitivity = 29.7% ). Level of significance is p<0.05 OR: odds ratio, CI: confidence interval

Explanatory variables (predictors)	B	Sig. (p-value)	OR	95% CI for OR	
				Lower	Upper
Family size	-0.15	0.04	0.86	0.74	0.99
Smoker or ex-smoker	1.84	<0.01>	6.29	2.69	14.68
Sleep disorders	1.45	<0.01>	4.25	2.06	8.77

## Discussion

This study aimed to assess the prevalence of substance use, identify associated risk factors, explore potential complications within this specific demographic, and investigate the relationship between sociodemographic and lifestyle variables. It is an addition to the growing literature on substance use and misuse in young adults in the region. Our results show that 20.2% of students in health colleges in Saudi Arabia have a lifetime history of substance use. Notably, there was no significant difference in substance use between male and female college students in our sample. This is in contrast to multiple other similar studies, both in the region and internationally, showing a higher proportion of males reporting substance use [3,10,11]. The reason behind this could be an increase in female substance use concurrent with rapid changes in social norms and habits or a selection bias, especially since significantly more females responded to our survey.

Our finding that smoking habits are correlated with substance use and are predictive of it aligns with the existing literature [4,12,13]. Indeed, this association is in agreement with the long-held belief that tobacco smoking can be a gateway to the use of other psychoactive substances in young individuals. The present results do not, however, demonstrate the previously reported association between alcohol use and other substance use. This could be attributed to the complete ban on alcohol by law in Saudi Arabia, which limited its availability to our surveyed young adults. Legal, religious, and cultural context frequently influences substance usage patterns. Our data suggest that the limited availability of alcohol may have contributed to the lack of relationship between alcohol and other substance use in Saudi Arabia, where alcohol is illegal and strictly enforced due to religious and cultural reasons.

Among the significant findings in our study is the observed association between lower GPAs and substance use. Students with lower GPAs may turn to substances as a way to cope with the academic pressure to enhance performance, which can make their academic difficulties worse. On the other hand, substance abuse can worsen motivation, attendance, and cognitive function, which further contributes to academic decline. A similar correlation has been reported with respect to sedative drug use in medical students in Saudi Arabia [14] and the misuse of other drugs in the United States, where lower GPAs were found to correlate with higher rates of misuse. The significant relationship between lower GPA and substance misuse can be attributed to various factors, such as increased stress or desperate attempts to improve academic performance. Alternatively, substance misuse could be the cause behind lower GPAs through its effects on attendance rates, cognition, or motivation [15].

The present analysis highlights the significant association between sleep and substance use in young adults. Our results demonstrated a correlation between substance use and reporting a history of sleep disorder, longer time needed to fall asleep, and daytime or unpredictable sleep habits. Sleep difficulties have been known to correlate with substance misuse [16,17]. Alcohol use and cigarette smoking have been especially implicated in causing sleep disturbances through various alterations in sleep architecture [18-21]. It is unfortunate that one of the most common justifications for substance use in our survey was mental health reasons since the literature shows that substance use would worsen sleep and mental health outcomes. Substance abuse is frequently attributed to mental health conditions such as depression and anxiety. At first, these drugs might offer temporary relief from their mental health problems, but drug abuse has the potential to worsen mental health issues over time, leading to a vicious cycle. Substance abuse can also interfere with regular sleep cycles, which exacerbates mental health issues and increases the need for drugs as a coping mechanism.

Our study was limited by the utilized convenience sampling method, which may not represent the target population. In addition, the majority of our respondents were female, limiting the generalizability of our results. Finally, the data was collected using a self-administered questionnaire.

## Conclusions

Although this topic has been investigated locally and globally, our cross-sectional national study showed that substance use is common among students in health colleges and is associated with smoking and sleep problems. These findings shed light on the unique dynamics around substance use in college students, including its relationship with academic performance. The survey could have limitations such as recall bias or selection bias, especially since significantly more females responded to our survey and the actual prevalence could be higher. Despite this, the study does provide important current data on substance use among students in health colleges. Our results show that there is a need for tailored interventions and comprehensive strategies to address the issues, ultimately fostering a healthier and more productive academic environment for students in health colleges. Also, the research presents insights and opportunities for future studies to identify the reasons behind substance misuse, its consequences on the well-being of future healthcare practitioners, and its potential impact on public health and safety.
